# A comprehensive evaluation of pre- and post-processing sperm parameters for predicting successful pregnancy rate following intrauterine insemination with the husband’s sperms

**DOI:** 10.1186/s12884-022-05029-8

**Published:** 2022-09-12

**Authors:** Yumei Luo, Mingxing Liu, Shunhong Wu, Mimi Zhang, Jingru Yuan, Yufang Zhong, Qing Li, Xiaofang Sun, Xia Xu, Detu Zhu

**Affiliations:** 1 Department of Obstetrics and Gynecology, Key Laboratory for Major Obstetric Diseases of Guangdong Province, The Third Affiliated Hospital of Guangzhou Medical University, Guangzhou, 510150 China; 2 Key Laboratory of Reproduction and Genetics of Guangdong Higher Education Institutes, The Third Affiliated Hospital of Guangzhou Medical University, Guangzhou, 510150 China; 3 Guangzhou Key Laboratory for Clinical Rapid Diagnosis and Early Warning of Infectious Diseases, Kingmed School of Laboratory Medicine, Guangzhou Medical University, Guangzhou, 510182 China

**Keywords:** Assisted reproduction technology, Intrauterine insemination, Artificial insemination by husband, Semen processing, Density gradient centrifugation, Sperm parameters, Clinical pregnancy rate, Logistic regression

## Abstract

**Background:**

To determine the predictive values of sperm parameters pre- and post-processing by density gradient centrifugation for clinical pregnancy rates (CPRs) following artificial insemination by husband (AIH) in infertile Chinese couples.

**Methods:**

A total of 3,522 AIH cycles from 1,918 couples were retrospectively analyzed. The parameters were compared between the pregnant and non-pregnant groups and further between different etiological groups (Male-factor, Both-male-and-female-factor, and Other-factor). Multivariate logistic regression analysis was performed to create models for predicting the CPRs of each etiological group.

**Results:**

The overall CPR was 13.3%. There were significant improvements for most sperm parameters after DGC. Multivariate logistic regression analysis indicated that, in overall AIH cases, the top parameters significantly influencing the CPR of AIH were pre-STR (OR = 1.037; *P* = 0.048) and post-VSL (OR = 1.036; *P* = 0.011). In the Male-factor Group, the top influencing parameters were pre-VCL (OR = 2.096; *P* = 0.008), pre-LIN (OR = 1.930; *P* = 0.002) and post-VSL (OR = 1.316; *P* = 0.023). In the Both-factor Group, the top influencing parameters were pre-VCL (OR = 1.451; *P* = 0.008) and post-motility (OR = 1.218; *P* = 0.049). In the Other-factor Group, the top influencing parameters were pre-VAP (OR = 1.715; *P* = 0.024), pre-STR (OR = 1.20; *P* = 0.011) and post-VSL (OR = 1.04; *P* = 0.017). Moreover, receiver operating characteristic analysis showed that the logistic regression models of the Male- and Both-factor Groups had greater powers for prognostic classification than those of other groups.

**Conclusions:**

This study demonstrated that some sperm parameters have a collinearity relationship in predicting the CPR following AIH. Moreover, the predictive capacity of a multivariate logistic regression model is better than those of individual parameters, especially for the Male- and Both-factor Groups. In these cases, pre-VCL is the common top influencing factor.

## Background

Owing to its minimal invasiveness, simple manipulation and low cost, intrauterine insemination (IUI) is currently the first-line assisted reproduction treatment for infertile couples [1]. IUI can be classified into artificial insemination by husband (AIH) and artificial insemination by donor (AID) according to the source of sperms. Prior to AIH, semen must be processed to separate viable spermatozoa with normal morphology and motility from unfavourable debris, non-sperm cells, and dead or immotile spermatozoa. The most widely used method for semen processing is density gradient centrifugation (DGC) [2].

It is expected that DGC processing leads to significant improvements in most sperm parameters [3, 4]. A number of previous studies indicated that some semen parameters, including semen volume, sperm motility and sperm morphology, could predict the outcomes of IUI [5–7]. Further, some studies demonstrated that the amount of sperm recovered after semen processing was significantly related to the clinical pregnancy rate (CPR) of AIH [8]. Also, there were predictive values of post-processing total motile sperm count (TMSC) and the normal form rate on AIH success [9–12]. In contrast, some studies suggested that post-processing semen parameters are not greater than those pre-processing in predicting CPR following AIH [2, 13]. Some studies have shown that the TMSC did not have a predictive value for the CPR [14, 15]. Besides, there was a large volume of published studies indicating that the normal rate of sperm morphology had nothing to do with the success rate of IUI [6, 14, 16]. Therefore, the prognostic values of pre- and pos-processing sperm parameters for AIH outcome remain controversial.

To determine the prognostic values of sperm parameters pre- and post-DGC processing for predicting CPRs following AIH in infertile Chinese couples, our study has retrospectively analyzed 1,918 Chinese infertile couples with a total of 3,522 AIH treatment cycles in the Third Affiliated Hospital of Guangzhou Medical University from September 2018 to May 2020, and provided a comprehensive assessment on the predictive values of sperm parameters pre- and post-DGC processing for the CPR following AIH.

## Methods

### Subjects

This was a retrospective cohort study enrolling infertile Chinese couples that underwent AIH at the fertility clinic of the Third Affiliated Hospital of Guangzhou Medical University between September 2018 and May 2020 [7]. The study was approved by the Ethics Committee of the Third Affiliated Hospital of Guangzhou Medical University (Protocol no. 2018–142).

Before the enrollment, the patients were diagnosed with the causes of infertility, with necessary assays to elicit etiologies. Female patients had hysterosalpingograms, and men had semen analyses. Causes of infertility were grouped into: (1) male factors, (2) female factors (ovulatory dysfunction, cervical factors, immune factors, endometriosis, endometriosis after pelvic plastic surgery, etc.), (3) combined male and female factors, and (4) unexplained infertility. Demographic data such as the age of the couple, duration of infertility, semen parameters before and after sperm processing, and the AIH outcomes were extracted from the patients’ records. All pregnancies were confirmed by positive beta-human chorionic gonadotropin (β-hCG) in the serum 14 days after AIH. Exclusion criteria: (1) Those whose record writing is not standard. (2) Those whose semen sample was collected with an incorrect container, partially lost, or was sent for examination after more than 30 min. (3) Those had undergone antibacterial treatment. (4) Those with incomplete data regarding the pregnancy outcome or missing data on pre- and post-processing sperm parameters. Finally, a total of 3,522 AIH cycles from 1,918 infertile Chinese couples were retrospectively analyzed (Table 1).

**Table 1:** Patient demographics

	Pregnant Group (*n* = 468)	Non-pregnant Group (*n* = 3,054)	*p*-value
*Female age*	31.02 ± 3.74	31.37 ± 4.05	0.133
*Male age*	32.93 ± 4.36	33.29 ± 4.75	0.402
*Cause of infertility*			0.585
Male-factor	82 (17.5%)	595 (19.5%)	
Both-male-and-female-factor	65 (13.9%)	427 (14.0%)	
Other-factor	321 (68.6%)	2,032 (66.5%)	
*Type of infertility*			0.988
Primary	287 (61.3%)	1,874 (61.4%)	
Secondary	181 (38.7%)	1,180 (38.6%)	
*IUI cycle*			0.049
1	265 (56.6%)	1,659 (54.3%)	
2	143 (30.6%)	1,085 (35.5%)	
≥ 3	60 (12.8%)	310 (10.2%)	

### Semen collection

Semen processing was performed as previously described [7]. In brief, semen specimens were produced with masturbation in a collection room at the fertility clinic. The specimens were kept at 37℃ temperature and were examined within half an hour post collection. After complete liquefaction, all samples were evaluated in a blinded fashion by a qualified technician to prevent the interobserver variation based on WHO 2010 criteria.

### Semen analysis

For semen analysis, 10 μL of semen was transferred to a counting chamber (AIYX, Cat# AI-20–4) and analyzed by computer-assisted sperm analysis (CASA; Hamilton Thorne HTCasa II 1.10.3). The sperm concentration was optimized to be (2 ~ 50) × 10^6^/mL. At least 200 sperms from 5 analyzed fields were counted. Sperm parameters including concentration, motility, total progressive motile sperm count (TPMSC), curvilinear velocity (VCL), straight-line velocity (VSL), average path velocity (VAP), linearity (LIN), straightness (STR), beat cross frequency (BCF), and amplitude of lateral head displacement (ALH), sperm head area were reported.

### Semen treatment

The samples were then treated by DGC method. In brief, the gradient separation solution (Vitrolife) was prepared in test tube, the lower layer is 1 mL 90% separation solution, the upper layer is 1 mL 45% separation solution. After the semen samples were evenly mixed, 1 mL of semen was taken and placed above the density gradient, centrifuged at 3000 r/min for 20 min. Then remove most of the supernatant from the upper layer. The sperm was suspended in 5 mL sperm culture medium and gently blown, and then centrifuged at 2000 r/min for 5 min and the supernatant was removed. Finally, 0.5 mL semen was used for sperm suspension, and sperm concentration, vitality and other semen parameters were tested. Semen samples with TPMSC > 2 × 10 are accepted to go for AIH treatment.

### Artificial insemination by husband

AIH was performed as previously described [7]. In brief, after the bladder was emptied, the patient took the bladder lithotomy position, with the vulva washed by saline, and the vagina, cervix and fornix wiped by a large cotton swab. A 1-mL syringe and an artificial insemination tube were connected in the uterine cavity. Then 0.5 mL of the husband’s sperm suspension was aspirated and slowly placed in the uterine cavity through the tube. After the sperm suspension is slowly injected into the uterus for 3 to 5 s, the tube was slowly withdrawn, and the patient was kept in the position of lowering the head and hips for approximately 30 min.

### Statistical analysis

**Fig. 1:**
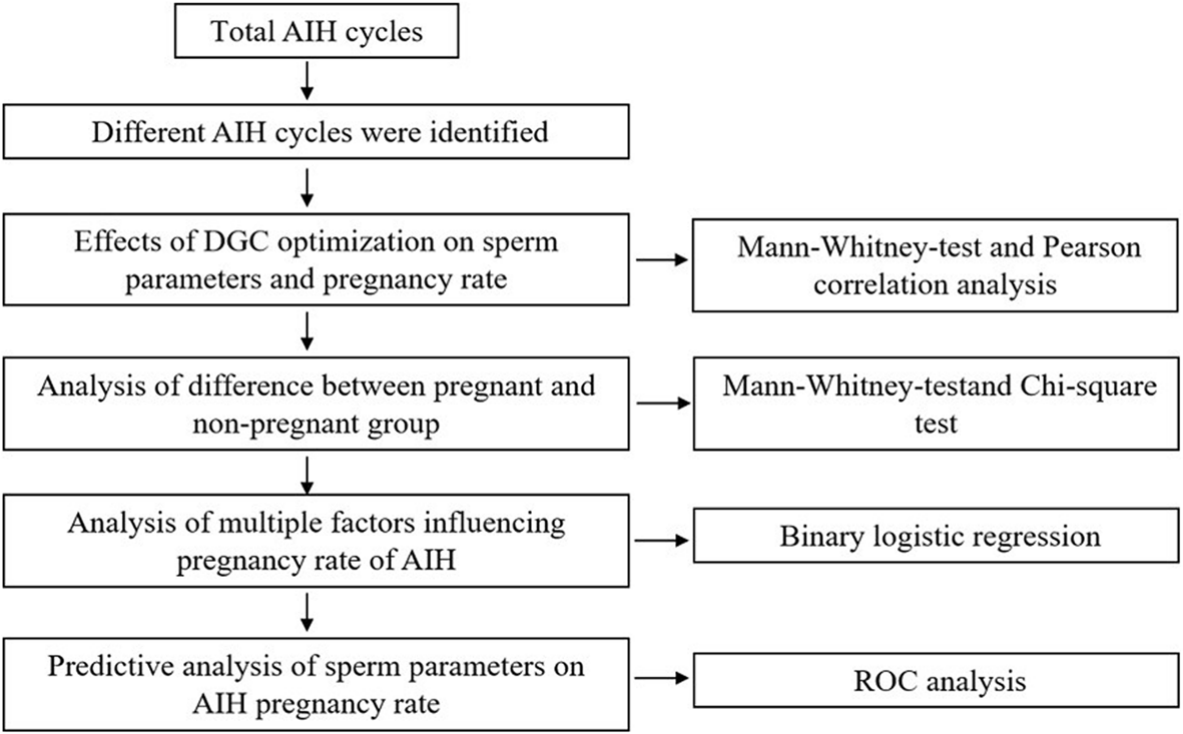
Experimental design

The SPSS v22.0 was used for statistical analysis [7]. The results are presented as mean ± standard deviation (SD). The analysis pipeline is shown in Fig. 1. Comparison of continuous variables between pregnant and non-pregnant groups was performed using the Mann–Whitney test because the data distribution was not normal according to the Kolmogorov–Smirnov test. Categorical variables were evaluated using the chi-square test. Spearman’s correlation coefficient analysis was performed to evaluate the correlation of absolute and relative changes of each variable and the CPR. Both categorical and continuous variables that might influence AIH pregnancy outcome were analyzed by backward binary multivariate logistic regression to identify predictive factors for different etiology groups. Receiver operating characteristic (ROC) curves were constructed to evaluate the prognostic classification capacities and calculate the clinically acceptable cut-off values for the logistic regression models of each etiology group. Statistical significance was accepted as *P* < 0.05.

## Results

### Sperm parameters were improved after semen processing

The semen samples were processed by DGC before the AIH treatments. Semen parameters before and after processing were compared across the pregnant and non-pregnant groups (Table 2). In both groups, there were significant improvements in most sperm parameters after DGC processing, except for TPMSC (all *P* < 0.001).

**Table 2:** Comparison of semen parameters before and after processing

Parameters	Pregnant group (*n* = 468)				Non-pregnant group (*n* = 3,054)			
	Before	After	(After-Before)	*p*-value	Before	After	(After-Before)	*p*-value
Concentration (× 10^6^/mL)	50.86 ± 31.47	71.14 ± 54.85	20.28 ± 37.72	**< 0.001**	52.21 ± 33.29	71.12 ± 54.49	18.91 ± 38.43	**< 0.001**
Motility (%)	61.69 ± 14.55	93.42 ± 7.56	31.73 ± 12.08	**< 0.001**	61.29 ± 14.07	93.43 ± 7.89	32.14 ± 11.69	**< 0.001**
TPMSC (× 10^6^/mL)	75.04 ± 63.50	32.97 ± 26.79	-42.07 ± 37.64	**< 0.001**	75.60 ± 63.88	33.08 ± 26.60	-42.52 ± 37.99	**< 0.001**
VAP (µm/s)	7.82 ± 3.84	24.47 ± 5.78	16.65 ± 6.67	**< 0.001**	7.86 ± 3.69	23.94 ± 5.83	16.08 ± 6.70	**< 0.001**
VSL (µm/s)	4.42 ± 2.26	14.10 ± 3.75	9.69 ± 4.14	**< 0.001**	4.42 ± 2.14	13.66 ± 3.57	9.24 ± 4.04	**< 0.001**
VCL (µm/s)	14.32 ± 5.87	47.39 ± 10.38	33.07 ± 11.56	**< 0.001**	14.37 ± 5.72	46.35 ± 10.69	31.98 ± 11.82	**< 0.001**
ALH (µm/s)	4.16 ± 0.50	6.56 ± 0.79	2.40 ± 0.91	**< 0.001**	4.16 ± 0.49	6.49 ± 0.80	2.33 ± 0.92	**< 0.001**
BCF (Hz)	5.45 ± 1.20	7.24 ± 0.70	1.79 ± 1.36	**< 0.001**	5.43 ± 1.15	7.25 ± 0.75	1.81 ± 1.33	**< 0.001**
STR (%)	46.73 ± 9.05	72.54 ± 7.86	65.17 ± 19.48	**< 0.001**	46.43 ± 8.41	71.95 ± 8.75	65.55 ± 18.48	**< 0.001**
LIN (%)	34.32 ± 6.28	47.50 ± 5.15	13.18 ± 7.59	**< 0.001**	34.18 ± 5.81	47.19 ± 5.72	13.01 ± 7.84	**< 0.001**
Sperm head area (µm^2^)	5.08 ± 0.48	5.55 ± 0.58	0.47 ± 0.70	**< 0.001**	5.12 ± 0.49	5.55 ± 0.57	0.43 ± 0.72	**< 0.001**

*Abbreviations*: *TPMSC* Total progressive motile sperm count, *VCL* Curvilinear velocity, *VSL* Straight-line velocity, *VAP* Average pathway velocity, *LIN* Linearity, *STR* Straightness, *BCF* Beat cross frequency, *ALH* Amplitude of lateral head displacement

To evaluate the impacts of these changes on the AIH outcomes, the correlations between the change values of each parameter and the likelihood of pregnancy were analyzed by Pearson correlation coefficients (Table 3). The results showed that neither absolute changes nor relative changes of the parameters, including the decrease of TPMSC, had any correlation with the AIH pregnancy outcome.

**Table 3:** Correlations between the changes of sperm parameters and overall AIH pregnancy rate

Parameters	absolute changes vs. overall CPR		Relative changes vs. overall CPR	
	PCC r	*p*-value	PCC r	*p*-value
Concentration (× 10^6^/mL)	0.017	0.307	0.026	0.122
Motility (%)	− 0.011	0.506	− 0.012	0.475
TPMSC (× 10^6^/mL)	0.003	0.871	0.019	0.259
VAP (µm/s)	0.019	0.248	0.019	0.261
VSL (µm/s)	0.027	0.103	0.024	0.162
VCL (µm/s)	0.025	0.135	0.02	0.234
ALH (µm/s)	0.020	0.227	0.019	0.249
BCF (Hz)	− 0.007	0.687	− 0.006	0.740
STR (%)	0.010	0.566	− 0.006	0.726
LIN (%)	0.008	0.634	0.008	0.620
Sperm head area (µm^2^)	0.022	0.190	0.023	0.175

*Abbreviations*: *CPR* clinical pregnancy rate, *PCC* Pearson correlation coefficient, *TPMSC* total progressive motile sperm count, *VCL* curvilinear velocity, *VSL* Straight-line velocity, *VAP* Average pathway velocity, *LIN* linearity, *STR* Straightness, *BCF* Beat cross frequency, *ALH* Amplitude of lateral head displacement

### Both pre- and post-processing semen parameters were not correlated with the pregnancy outcome of overall AIH cases

The pre- and post- processing semen parameters and sperm motion kinetic parameters (Table 4), and pre-processing sperm morphological parameters (Table 5) were compared between the Pregnant (*n* = 468) and Non-pregnant (*n* = 3,054) Groups of overall AIH cases. The results showed that these parameters were not significantly different between the two groups. It was probably due to the confounding effects of influencing factors other than the male factors.

**Table 4:** Comparison of semen parameters before and after processing between pregnant and non-pregnant groups

Parameters	Before semen treatment				After semen treatment	
	Pregnant (*n* = 468)	Non-pregnant (*n* = 3,054)	*p*-value	Pregnant (*n* = 468)	Non-pregnant (*n* = 3,054)	*p*-value
Concentration (× 10^6^/mL)	50.86 ± 31.47	52.21 ± 33.29	0.468	71.14 ± 54.85	71.12 ± 54.49	0.896
Motility (%)	61.69 ± 14.55	61.29 ± 14.07	0.546	93.42 ± 7.56	33.08 ± 26.60	0.582
TPMSC (× 10^6^/mL)	75.04 ± 63.50	75.60 ± 63.88	0.986	93.42 ± 7.56	93.43 ± 7.89	0.983
VAP (µm/s)	7.82 ± 3.84	7.86 ± 3.69	0.703	24.47 ± 5.78	23.94 ± 5.83	0.213
VSL (µm/s)	4.42 ± 2.26	4.42 ± 2.14	0.751	14.10 ± 3.75	13.66 ± 3.57	0.105
VCL (µm/s)	14.32 ± 5.87	14.37 ± 5.72	0.779	47.39 ± 10.38	46.35 ± 10.69	0.107
ALH (µm/s)	4.16 ± 0.50	4.16 ± 0.49	0.698	6.56 ± 0.79	6.49 ± 0.80	0.192
BCF (Hz)	5.45 ± 1.20	5.43 ± 1.15	0.681	7.24 ± 0.70	7.25 ± 0.75	0.415
STR (%)	46.73 ± 9.05	46.43 ± 8.41	0.64	72.54 ± 7.86	71.95 ± 8.75	0.214
LIN (%)	34.32 ± 6.28	34.18 ± 5.81	0.837	47.50 ± 5.15	47.19 ± 5.72	0.299
Sperm head area (µm^2^)	5.08 ± 0.48	5.12 ± 0.49	0.086	5.55 ± 0.58	5.55 ± 0.57	0.743

*Abbreviations*: *TPMSC* total progressive motile sperm count, *VCL* curvilinear velocity, *VSL* straight-line velocity, *VAP* average pathway velocity, *LIN* Linearity; *STR* Straightness, *BCF* Beat cross frequency, *ALH* Amplitude of lateral head displacement

**Table 5:** Comparison of sperm morphological parameters between pregnant and non-pregnant groups

Parameters	Pregnant (*n* = 468)	Non-pregnant (*n* = 3,054)	*p*-value
SDI	1.24 ± 0.11	1.24 ± 0.11	0.44
TZI	1.30 ± 0.10	1.30 ± 0.10	0.718
H (%)	94.69 ± 7.54	94.79 ± 8.64	0.328
M (%)	16.47 ± 5.23	16.57 ± 5.08	0.559
P (%)	5.15 ± 3.12	5.48 ± 3.31	0.076
C (%)	6.83 ± 4.74	6.84 ± 4.81	0.917
Normal forms (%)	4.82 ± 4.24	4.42 ± 3.79	0.141
Abnormal forms (%)	95.17 ± 4.24	95.58 ± 3.79	0.113

*Abbreviations*: *SDI* Sperm deformity index, *TZI* Teratozoospermia index, *H* Sperm headpiece deformity, *M* Sperm middle piece deformity, *P* Sperm principal piece deformity, *C* Sperm cytoplasm deformity

### Comparison of semen parameters between different etiological groups of AIH

Assuming that the above semen parameters might be more relevant with male factors, we classified the AIH cases into Male-factor (*n* = 677), Both-male-and-female-factor (*n* = 492) and Other-factor (*n* = 2,353) Groups by etiologies. The comparison of the parameters among these three groups was performed using the Kruskal–Wallis test because data distribution was not normal according to the Kolmogorov–Smirnov test.

Our results showed that all pre-processing semen parameters and sperm motion kinetic parameters of the Other-factor Groups were significantly higher than those in the Male- and Both-factor Groups (Table 6); meanwhile, all pre-processing sperm deformity parameters except the sperm cytoplasm deformity of the Other-factor Groups were significantly lower than those in the Male- and Both-factor Groups (Table 7) (all *P* < 0.01). This was reasonable as the pre-processing sperm quality of the cases due to non-male factors should be better than those due to or partly due to male factors.

**Table 6:** Comparison of semen parameters before and after processing between different etiological groups

Parameters	Before sperm processing				After sperm processing			
	Male-factor (*n* = 677)	Both-factor (*n* = 492)	Other-factor (*n* = 2,353)	*p*-value	Male-factor (*n* = 677)	Both-factor (*n* = 492)	Other-factor (*n* = 2,353)	*p*-value
Concentration (× 10^6^/mL)	44.02 ± 31.41^a^	44.81 ± 29.91^a^	55.85 ± 33.48^b^	**< 0.001**	53.45 ± 47.32^a^	56.11 ± 45.65^a^	79.35 ± 56.28^b^	**< 0.001**
Motility (%)	54.95 ± 14.31^a^	57.29 ± 14.14^b^	64.03 ± 13.26^c^	**< 0.001**	90.26 ± 10.31^a^	91.45 ± 9.29^a^	94.75 ± 6.17^b^	**< 0.001**
TPMSC (× 10^6^/mL)	54.81 ± 53.70^a^	59.03 ± 55.44^b^	84.94 ± 66.01^c^	**< 0.001**	24.06 ± 22.97^a^	25.69 ± 22.28^a^	37.20 ± 27.45^b^	**< 0.001**
VAP (µm/s)	6.55 ± 3.53^a^	6.80 ± 3.45^a^	8.45 ± 3.68^b^	**< 0.001**	23.66 ± 6.03	23.49 ± 5.61	24.23 ± 5.80	0.113
VSL (µm/s)	3.69 ± 2.08^a^	3.84 ± 2.00^a^	4.74 ± 2.14^b^	**< 0.001**	13.46 ± 3.75	13.46 ± 3.35	13.84 ± 3.60	0.231
VCL (µm/s)	12.45 ± 5.38^a^	12.82 ± 5.37^a^	15.24 ± 5.71^b^	**< 0.001**	45.96 ± 11.04^ab^	45.42 ± 10.37^a^	46.87 ± 10.58^b^	**0.018**
ALH (µm/s)	3.99 ± 0.46^a^	4.03 ± 0.46^a^	4.24 ± 0.49^b^	**< 0.001**	6.46 ± 0.84	6.42 ± 0.78	6.53 ± 0.80	0.128
BCF (Hz)	4.97 ± 1.17^a^	5.11 ± 1.15^a^	5.64 ± 1.09^b^	**< 0.001**	7.16 ± 0.78^a^	7.28 ± 0.72^ab^	7.26 ± 0.74^b^	**0.025**
STR (%)	43.24 ± 8.29^a^	44.21 ± 8.18^a^	47.87 ± 8.26^b^	**< 0.001**	71.32 ± 9.02	72.05 ± 8.38	72.23 ± 8.57	0.104
LIN (%)	31.88 ± 5.67^a^	32.60 ± 5.59^a^	35.20 ± 5.73^b^	**< 0.001**	46.74 ± 5.85	47.32 ± 5.50	47.36 ± 5.62	0.056
Sperm head area (µm^2^)	5.27 ± 0.57^a^	5.20 ± 0.50^a^	5.05 ± 0.44^b^	**< 0.001**	5.61 ± 0.61^a^	5.57 ± 0.56^ab^	5.53 ± 0.57^b^	**0.002**

Values on the same row with different superscripts are significantly different (*P* < 0.05)

*Abbreviations*: *TPMSC* Total progressive motile sperm count, *VCL* Curvilinear velocity, *VSL* Straight-line velocity, *VAP* Average pathway velocity, *LIN* Linearity, *STR* Straightness, *BCF* Beat cross frequency, *ALH* Amplitude of lateral head displacement

**Table 7:** Comparison of sperm morphological parameters between different etiology groups

Parameters	Male-factor (*n* = 677)	Both-factor (*n* = 492)	Other-factor (*n* = 2,353)	*p*-value
SDI	1.27 ± 0.11^a^	1.28 ± 0.11^a^	1.22 ± 0.11^b^	**< 0.001**
TZI	1.31 ± 0.11^ab^	1.31 ± 0.10^a^	1.30 ± 0.10^b^	**0.009**
H (%)	97.02 ± 6.95^a^	97.32 ± 5.16^a^	93.60 ± 9.19^b^	**< 0.001**
M (%)	17.23 ± 5.62^a^	17.52 ± 5.05^a^	16.17 ± 4.91^b^	**< 0.001**
P (%)	5.64 ± 3.36^a^	5.91 ± 3.36^a^	5.28 ± 3.23^b^	**< 0.001**
C (%)	7.19 ± 5.29	7.02 ± 5.01	6.70 ± 4.60	0.364
Normal forms (%)	2.48 ± 2.49^a^	2.38 ± 2.58^a^	5.49 ± 3.99^b^	**< 0.001**
Abnormal forms (%)	97.52 ± 2.49^a^	97.62 ± 2.58^a^	94.51 ± 4.00^b^	**< 0.001**

Values on the same row with different superscripts are significantly different (*P* < 0.05)

*Abbreviations*: *SDI* Sperm deformity index, *TZI* Teratozoospermia index, *H* Sperm headpiece deformity, *M* Sperm middle piece deformity, *P* Sperm principal piece deformity, *C* Sperm cytoplasm deformity

Nevertheless, in post-processing parameters, only the sperm concentration, motility, TPMSC, VCL and BCF parameters of the Other-factor Group were significantly higher than those of the Male- and Both-factor Groups (Table 6) (all *P* < 0.05), reflecting an improvement of sperm quality by the DGC processing.

Our results showed that most pre- and post-processing semen parameters were significantly different among the etiological groups; hence, it was necessary to count in the etiology when analyzing the influence of semen parameters on AIH pregnancy.

### Multivariate logistic regression analysis of the influencing factors for different etiological groups of AIH

Variables that might have impacts on pregnancy outcome were included in the logistic regression analysis, with CPRs of overall AIH cases and different etiological groups as the dependent variables, and pre-processing sperm morphological parameters, pre- and post- processing semen parameters and sperm motion kinetic parameters as independent variables. Then multivariate binary logistic regression analysis (backward conditional) was performed.

The results showed that, in overall cases, after excluding confounding factors, the top influencing factors were pre-STR (OR = 1.037; 95% CI: 1.000–1.076; *P* = 0.048) and post-VSL (OR = 1.036; 95% CI: 1.008–1.064; *P* = 0.011) (Table 8). Further, in the Male-factor Group, the top influencing factors were pre-VCL (OR = 2.096; 95% CI: 1.218–3.607; *P* = 0.008); pre-LIN (OR = 1.930; 95% CI: 1.276–2.919; *P* = 0.002) and post-VSL (OR = 1.316; 95% CI: 1.039–1.666; *P* = 0.023) (Table 9). In the Both-factor Group, the top influencing factors were pre-VCL (OR = 1.451; 95% CI: 1.104–1.906; *P* = 0.008) and post-motility (OR = 1.218; 95% CI: 1.000–1.484; *P* = 0.049) (Table 10). In the Other-factor Group, the top influencing factors were pre-VAP (OR = 1.715; 95% CI: 1.073–2.740; *P* = 0.024); pre-STR (OR = 1.20; 95% CI: 1.042–1.382; *P* = 0.011) and post-VSL (OR = 1.04; 95% CI: 1.007–1.074; *P* = 0.017) (Table 11).

**Table 8:** Influencing factors for overall AIH pregnancy identified by multivariate logistic regression analysis

Parameters	β	Odd Ratio (95% CI)	*p*-value
pre-VSL	-0.155	0.856 (0.740–0.991)	**0.037**
pre-STR	0.037	1.037 (1.000–1.076)	**0.048**
post-VSL	0.035	1.036 (1.008–1.064)	**0.011**
P	-0.027	0.973 (0.943–1.004)	0.086
Abnormal forms	-0.017	0.983 (0.966–1.001)	0.061
Constant	-1.65	0.192	0.125

*Abbreviations*: *VSL* Straight-line velocity, *STR* Straightness, *P* Sperm principal piece deformity, *β* Regression coefficient, *CI* Confidence interval

**Table 9:** Influencing factors for AIH pregnancy of the Male-factor Group identified by multivariate logistic regression analysis

Parameters	β	Odd Ratio (95% CI)	*p*-value
pre-VAP	-0.707	0.493 (0.247–0.984)	**0.045**
pre-VSL	-1.765	0.171 (0.045–0.658)	**0.01**
pre-VCL	0.74	2.096 (1.218–3.607)	**0.008**
pre-BCF	-1.487	0.226 (0.084–0.611)	**0.003**
pre-LIN	0.657	1.93 (1.276–2.919)	**0.002**
pre-Sperm head area	-0.585	0.557 (0.294–1.056)	0.073
post-VAP	-0.117	0.89 (0.797–0.993)	**0.037**
post-VSL	0.275	1.316 (1.039–1.666)	**0.023**
post-BCF	0.483	1.621 (0.991–2.652)	0.054
post-STR	-0.062	0.939 (0.878–1.005)	0.071
H	0.354	1.424 (0.808–2.513)	0.222
Normal forms	0.476	1.61 (0.899–2.883)	0.109
Constant	-46.353	0	0.116

*Abbreviations*: *VCL* Curvilinear velocity, *VSL* Straight-line velocity, *VAP* Average pathway velocity, *LIN* Linearity, *STR* Straightness, *BCF* Beat cross frequency, *H* Sperm headpiece deformity, *β* Regression coefficient, *CI* Confidence interval

**Table 10:** Influencing factors for AIH pregnancy of the Both-factor Group identified by multivariate logistic regression analysis

Parameters	β	Odd Ratio (95% CI)	*p*-value
pre-VAP	-0.634	0.53 (0.34–0.827)	**0.005**
pre-VCL	0.372	1.451 (1.104–1.906)	**0.008**
post-motility	0.197	1.218 (1.000–1.484)	**0.049**
post-VCL	0.186	1.204 (0.995–1.458)	0.056
post-ALH	-2.209	0.11 (0.008–1.431)	0.092
P	-0.102	0.903 (0.826–0.988)	**0.026**
Constant	-1.054	0.349	0.828

*Abbreviations*: *VCL* Curvilinear velocity, *VAP* Average pathway velocity, *ALH* Amplitude of lateral head displacement, *P* Sperm principal piece deformity, *β* Regression coefficient, *CI* Confidence interval

**Table 11:** Influencing factors for AIH pregnancy of the Other-factor Group identified by multivariate logistic regression analysis

Parameters	β	Odd Ratio (95% CI)	*p*-value
pre-VAP	0.539	1.715 (1.073–2.74)	**0.024**
pre-VCL	-0.342	0.71 (0.537–0.94)	**0.017**
pre-STR	0.182	1.2 (1.042–1.382)	**0.011**
pre-LIN	-0.283	0.754 (0.601–0.945)	**0.014**
post-VSL	0.039	1.04 (1.007–1.074)	**0.017**
Constant	-0.528	0.59	0.602

*Abbreviations*: *VAP* Average pathway velocity, *VCL* Curvilinear velocity, *VSL*, Straight-line velocity, *LIN* Linearity, *STR* Straightness, *β* Regression coefficient, *CI* Confidence interval

**Fig. 2:**
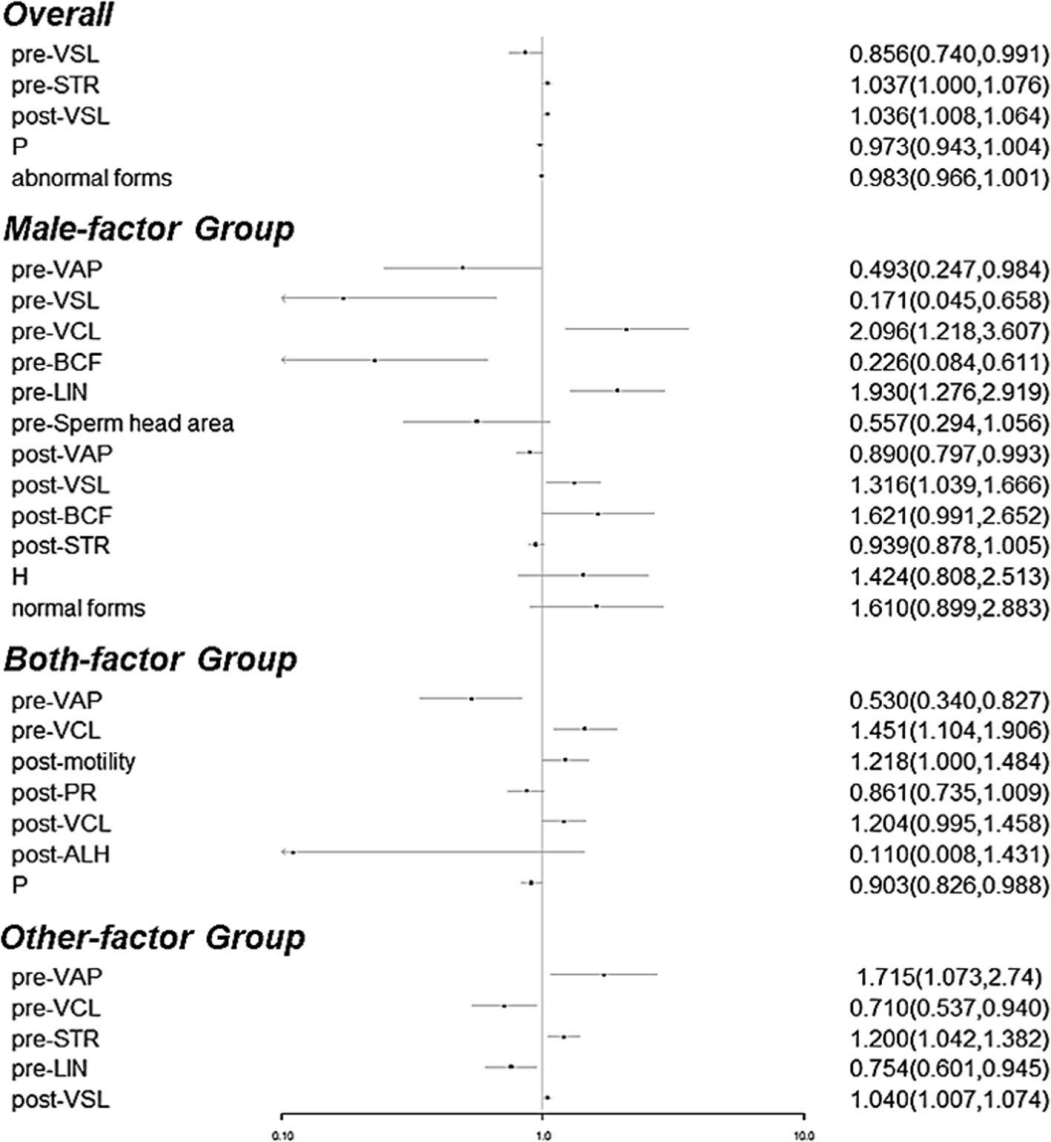
Forest plot for the influencing factors of overall AIH cases and different etiological groups identified by multivariate logistic regression analysis

Taken together, most of the significantly influencing factors were sperm motion kinetic parameters. Moreover, there were collinearity relationships among these parameters affecting the AIH outcomes in different etiological groups (Fig. 2).

### Semen parameters pre- and post-processing had greater prognostic powers in Male- and Both-factor Groups of AIH

The expected probability equations of overall AIH cases and of each etiological group created by the multivariate logistic regression models were further evaluated with ROC curves as predictors for AIH pregnancy. In overall cases, the cut-off value of the logistic regression equation was 14.77% (Sensitivity: 32.7%; Specificity:77.7%). For comparison, the cut-off values of the logistic regression equations of the Male-factor, Both-factor and Other-factor Groups were 12.46% (Sensitivity: 59.8%; Specificity: 65.7%), 8.74% (Sensitivity: 90.8%; Specificity: 33.0%) and 14.82% (Sensitivity: 35.8%; Specificity: 73.2%), respectively (Table 12).

**Table 12:** ROC analysis for multivariate logistic regression models of overall AIH cases and different etiology groups

Multivariate logistic regression models	AUC	95% CI	p-value	Cut-off	Sensitivity	Specificity
Overall	0.551 ± 0.014	0.523–0.58	**< 0.001**	14.77	32.7	77.7
Male-factor Group	0.668 ± 0.031	0.606–0.729	**< 0.001**	12.46	59.8	65.7
Both-male-and-female-factor Group	0.655 ± 0.034	0.588–0.722	**< 0.001**	8.74	90.8	33.0
Other-factor Group	0.544 ± 0.017	0.51–0.578	**0.011**	14.82	35.8	73.2

*Abbreviations*: *ROC* Receiver-operating characteristic, *AUC* Area under curve, *CI* Confidence interval

**Fig. 3:**
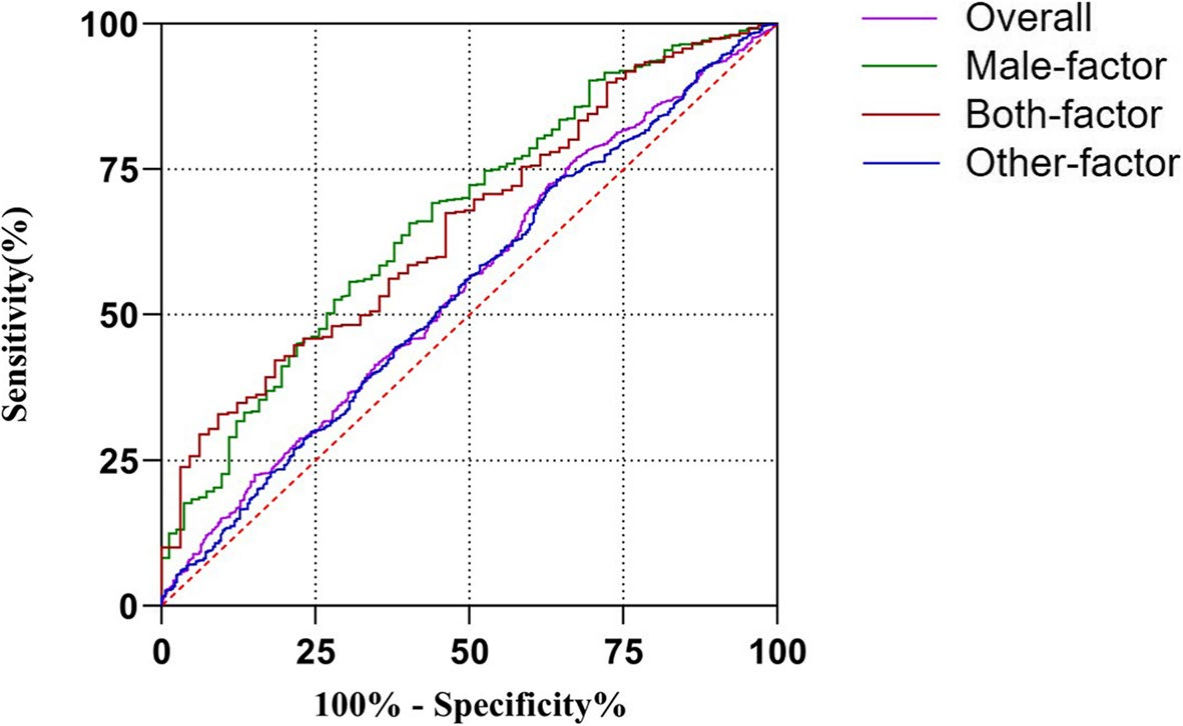
ROC curves for the multivariate logistic regression models of overall AIH cases and different etiological groups

The areas under ROC curves (AUC) of overall cases, the Male-factor, Both-factor and Other-factor Groups were 0.551, 0.668, 0.655 and 0.544, respectively (Table 12; Fig. 3). Therefore, the prognostic classification capacities of the logistic regression equations for the Male-factor and Both-factor Groups were greater than those for the other groups. And pre-VCL is the common top influencing factor in these two groups.

## Discussion

Although the AIH technology has the advantages of simple operation, low cost, high safety, and low invasiveness, a major problem with AIH treatment is the lower pregnancy rate (about 5.0 to 15.0%). In this study, 3,522 AIH cycles were achieved from 1,918 couples, resulting in a CPR of 13.3%, which is comparable to those of other reports [17–19]. In recent years, there has been an increasing amount of literature on AIH; however, there has always been controversy about the factors that influence the outcome of AIH. Therefore, it is particularly important to explore the factors that affect AIH pregnancy and take corresponding interventions to improve the CPR. This study aimed to evaluate the prognostic capacities of semen parameters before and after sperm selection processing on AIH outcomes.

To select high-quality sperms for AIH, the most widely used methods for semen treatment are DGC and swim-up. As instructed by the WHO, these treatment methods should be chosen according to the characteristics of the samples [20]. It was suggested to select swim-up in cases with normal sperms, while DGC for other cases, as the latter resulted in higher recovery rates [21]. Significantly higher CPRs were observed among couples with unexplained infertility using DGC than those using swim-up [22]. Besides, Karamahmutoglu et al*.* found that in the cases of unexplained infertility, the DGC technique contributed to a significantly higher CPR compared to the swim-up technique as it could select sperms with better DNA and chromatin structures; however, in male subfertile patients, both techniques yield similar clinical outcomes [22]. Oguz et al*.* compared the two methods and found that the swim-up method significantly reduced sperm DNA fragmentation and mgiht have some prognostic value on IUI in patients with decreased sperm DNA integrity [23]. Recently, there is a new sperm selection method—microfluid sperm sorting chip [24], which adopts microfluidic devices [25–27] for sperm selection. It would be of interest to investigate the influences of different semen processing methods, especially including the newly developed methods, on CPRs in future studies, so as to choose a better method for assisted reproduction treatment [3]. In this study, our results showed that DGC processing led to significant increases in most sperm parameters, except for TPMSC (Table 1); however, the decrease in TPMSC did not affect the pregnancy outcome of AIH (Table 3).

There was a large volume of published studies describing the impacts of semen parameters on the success of IUI [28, 29]. It has been reported that sperm kinetic parameters of motility, including VAP, VSL, VCL, ALH and LIN, are all associated with fertility [4, 30–32]. Our team’s previous research has shown that cycle treatment options, single/double IUI, female age, sperm VSL, SDI, and normal form rate could predict successful pregnancy following AIH in China. The multivariate logistic regression equation exhibited a greater value for prognostic classification than single predictors [7]. Similarly, our logistic regression analysis showed that there were some sperm kinetic parameters of motility could affect AIH outcomes, though the effects were varied between different etiological groups (Table 4). Besides, some researchers ascertained that the total number of active sperm and concentration are related to pregnancy [31, 33]. However, our study demonstrated that semen volume, sperm concentration and TPMSC before and after processing had no significant effect on AIH success.

A growing body of evidences is showing the impacts of sperm morphological parameters on the successful rate of AIH. Several studies advised couples with ≤ 4% normal sperm form to choose IVF or ICSI instead of IUI [5, 34]. Erdem et al*.* pointed out that the predictive value of morphological assessment in unexplained infertility is not reliable; however, in male subfertility, normal sperm form > 4.5% after processing could increase the CPR [35]. Louise et al*.* also stated that normal sperm form ≤ 4% is more important in couples with male infertility factors [16]. Contrary to the above research, a recent review suggested that sperm morphological parameters had low predictive values for pregnancy success in both natural and assisted reproduction [36]. Kohn et al*.* also supported this conclusion and suggested that the current sperm morphology assessment is so strict that its predictive value for IUI has been lost [37]. In our study, the morphological parameters of different etiologies groups also had no significant influence on AIH pregnancy success (Table 5).

Semen parameters with predictive powers have been extensively exploited for prognostic classification of infertile patients. For example, Hamilton et al*.* indicated that TMSC had a predictive value for CPRs following IUI in infertile cases associated with male or unexplained factors, and considered TMSC > 5 × 10^6^ /mL as the optimal stratifying criterion [38]. Youn et al*.* demonstrated that the composition of semen parameters such as RAPID 30.1%, motility 51.4%, and concentration 111 × 10^6 ^/mL before sperm preparation could be useful in predicting IUI outcome [31]. Oppositely, some researchers illuminated that in the multivariable model, the predictive powers of sperm parameters were rather low [16, 36, 39], which might be due to a lack of prospective studies, a lack of standardization in semen testing methodology, and a huge heterogeneity of patient groups and IUI treatment strategies. More prospective cohort trials and prospective randomized trials investigating the predictive value of semen parameters on IUI outcome are urgently needed [40]. In our study, we classified the cases into different etiological groups – Male-factor, Both-male-and-female-factor and Other-factor. Our comparison analysis suggested that both pre- and post-processing parameters were quite different among the etiological groups (Tables 5 and 6); therefore, it is necessary to count in the etiology when analyzing the impacts of semen parameters on AIH pregnancy. Further logistic regression analysis showed that models integrating multiple influencing factors exhibited much better predictive abilities on AIH outcomes than individual parameters (Tables 7, 8, 9, 10; Fig. 2). ROC curve analysis indicated that the prognostic capacities of the logistic regression models were greater in the Male- and Both-factor Groups than in the overall and Other-factor Groups (Tables 12; Fig. 3). This was reasonable as infertility cases caused by non-male factors might be less impacted by the improvement of sperm quality.

There are still a few limitations in our current study. At present, the overall pregnancy rate of AIH is low. Due to various causes of infertility in both couples, the predictive value of a single predictor is low. Moreover, surgery needs to be performed immediately after sperm processing, which results in our study remaining in retrospective analysis. Further comprehensive studies are of need in the future.

In summary, our study showed that the DGC method prior to AIH significantly improved the sperm quality, but the change values were not correlated with the CPR. We found that some sperm parameters pre- or post-processing could predict the AIH outcome, and there was a collinearity relationship among these semen parameters. Moreover, we accredited that the prognostic capacities of multivariate logistic regression models were better than those of individual parameters, especially in cases caused or partly caused by male factors. In these cases, pre-processing VCL is the common top influencing factor.

## Data Availability

The datasets used in this study are not publicly available due to the clinical data management policy of the health authority but are available from the corresponding authors upon reasonable request.

## Abbreviations

CPR: Clinical pregnancy rate
PCC: Pearson correlation coefficient
TPMSC: Total progressive motile sperm count
VCL: Curvilinear velocity
VSL: Straight-line velocity
VAP: Average pathway velocity
LIN: Linearity
STR: Straightness
BCF: Beat cross frequency
ALH: Amplitude of lateral head displacement
SDI: Sperm deformity index
TZI: Teratozoospermia index
H: Sperm headpiece deformity
M: Sperm middle piece deformity
P: Sperm principal piece deformity
C: Sperm cytoplasm deformity
ROC: Receiver-operating characteristic
AUC: Area under curve
CI: Confidence interval
